# Asthma is associated with carotid arterial injury in children: The Childhood Origins of Asthma (COAST) Cohort

**DOI:** 10.1371/journal.pone.0204708

**Published:** 2018-09-27

**Authors:** Matthew C. Tattersall, Michael D. Evans, Claudia E. Korcarz, Carol Mitchell, Elizabeth Anderson, Douglas F. DaSilva, Lisa P. Salazar, James E. Gern, Daniel J. Jackson, Robert F. Lemanske, James H. Stein

**Affiliations:** 1 Department of Medicine, Division of Cardiovascular Medicine, University of Wisconsin School of Medicine and Public Health, Madison, Wisconsin, United States of America; 2 Department of Biostatistics and Medical Informatics, University of Wisconsin-Madison, Madison, Wisconsin, United States of America; 3 Department of Pediatrics, University of Wisconsin School of Medicine and Public Health, Madison, Wisconsin, United States of America; Medizinische Universitat Innsbruck, AUSTRIA

## Abstract

**Background:**

Asthma is associated with an increased cardiovascular disease (CVD) risk in adults, but the impact of asthma and atopic conditions on CVD risk in children is less well established. We hypothesized that children in the Childhood Origins of Asthma (COAST) Cohort with asthma and atopic conditions would have early carotid arterial injury.

**Methods:**

The COAST study is a longitudinal birth cohort of children at increased risk of developing asthma. Children underwent ultrasonography measuring far wall right carotid bifurcation (RCB) and common carotid artery (RCCA) intima-media thickness (IMT; a measure of arterial injury). Multivariable linear regression models adjusted for age, gender, race, blood pressure, and body-mass index were used to assess associations of asthma and markers of arterial injury.

**Results:**

The 89 participants were a mean (standard deviation) 15.3 (0.6) years old and 42% were female; 28 asthmatics had atopic disease, 34 asthmatics were without other atopic disease, and 15 non-asthmatics had atopic disease. This study population was compared to 12 controls (participants free of asthma or atopic disease). Compared to controls (589 μm), those with atopic disease (653 μm, p = 0.07), asthma (649 μm, p = 0.05), or both (677 μm, p = 0.005) had progressively higher RCB IMT values (p_trend_ = 0.011). In adjusted models, asthmatic and/or atopic participants had significantly higher RCB IMT than those without asthma or atopic disease (all p≤0.03). Similar relationships were found for RCCA IMT.

**Conclusion:**

Adolescents with asthma and other atopic diseases have an increased risk of subclinical arterial injury compared to children without asthma or other atopic disease.

## Introduction

Asthma and cardiovascular disease (CVD) pose significant public health burdens. Asthma is a highly prevalent condition afflicting over 25 million individuals in the United States.[1–2] CVD is the leading cause of death for adults in the United States.[3–4] Inflammation and immune activation are central in the pathophysiology of both asthma and CVD.[5–8] Prior studies have demonstrated increased CVD risk among individuals with other chronic inflammatory diseases [5,8–10].

We and others have demonstrated that in adults, asthma is associated with increased CVD risk, likely because of a common inflammatory pathophysiology [5,11–15]. Prior studies, however, were not performed in asthma-specific cohorts with well-characterized asthma subtypes; moreover, the adults that were studied had confounding CVD risk factors. To our knowledge, no prior study has investigated the associations among asthma, atopy, and subclinical CVD in a well-characterized asthma cohort of children.

The purpose of the present study was to investigate the associations among asthma, atopy and subclinical CVD in the Childhood Origins of Asthma (COAST) study, a birth cohort of children at increased risk for the development of asthma and atopic conditions. We hypothesized that the presence of asthma and atopic disease in children is associated with greater arterial injury manifested as a thicker carotid intima-media thickness.

## Methods

### Participants

The COAST study is an NHLBI funded observational birth cohort designed to investigate genetic and environmental factors contributing to the development of asthma in childhood [16]. To qualify for COAST enrollment, at least one parent was required to have respiratory allergies (defined as one or more positive aeroallergen skin tests) and/or a history of physician-diagnosed asthma [16]. A total of 289 newborns were enrolled from November of 1998 through May 2000; additional children with asthma meeting these same inclusion criteria were enrolled in the COAST study between 9 and 11 years of age. We recruited 90 participants that presented for an onsite annual COAST examination between August 2014 and August 2015; one had type I diabetes mellitus and was excluded from the analysis. Details on the design of COAST have been published previously.[16] Participant consent/assent was obtained at the time of their annual COAST examination. This study was approved by the University of Wisconsin Institutional Review Board. All COAST participants presenting for their annual onsite examination were eligible for the current study.

### Carotid ultrasonography

B-mode ultrasound images of the far walls of the right common carotid artery (RCCA) and right carotid artery bifurcation (RCB) were obtained by registered sonographers (Siemens Acuson S2000, Malvern, PA, USA) with a 9L4 transducer. Because of our participants’ young age, we needed to be sensitive to the duration of the ultrasound exam, so we imaged right carotid artery only, as in several prior studies [17–19]. The RCCA was defined as the distal 10 mm of the carotid artery proximal to the RCB. The proximal 10 mm of the RCB were measured after being exported in DICOM format to a syngo Ultrasound Workplace reading station (Siemens Medical, Malvern, PA). Maximum segmental IMT measurements were obtained from end-diastolic still frames using a semi-automated border detected program and averaging tracings from 3 distinct cardiac cycles (Arterial Health Package, Siemens Medical, Malvern, PA) for IMT measurement.[20–21]. The mean of the maximum measurements of the RCCA and the RCB were calculated [22–23]. Carotid IMT measurements are very reproducible in the UW Atherosclerosis Imaging Research Program (AIRP) lab, the intra-class correlation coefficients (ICC) for *intra*-reader reproducibility for mean CCA and bulb/ICA IMT are 0.98–0.99 in the UW AIRP. For scan-rescan variability, for 44 repeated studies, the Pearson correlation coefficient is 0.98 for maximum CCA IMT. Mean differences were 0.006 (0.034)– 0.0007 (0.0.036) mm. There were no outliers noted on limit of agreement analyses for matched segments [24–25].

### Asthma definition

In COAST, asthma assessments occurred at the end of the 6th, 8th, 11th, and 13th years of life. The presence of asthma at any of those exams qualified the participant as asthmatic for the study. A diagnosis of asthma in COAST has been previously reported and was based on the documented presence of one or more of the following characteristics in the previous year [26,27] (1) physician diagnosis of asthma, (2) use of physician-prescribed albuterol for coughing or wheezing episodes, (3) use of a daily controller medication, (4) step-up plan including use of albuterol or short-term use of inhaled corticosteroids during illness, or (5) use of prednisone for asthma exacerbation.

### Atopic disease definition

Atopic disease was defined as the presence of physician-diagnosed atopic dermatitis, eczematous skin conditions or allergic rhinitis. Atopic dermatitis was defined as documentation by a health care provider in the participant’s medical record. There were two participants with physician documented “possible” atopic dermatitis. For proper adjudication, a review of 3 additional forms administered at ages 5–8 was performed and included the following: a study physician questionnaire, a parent-report questionnaire, and the scheduled physical exam, all of these three forms had to be consistent with atopic dermatitis for these participants to be classified as “atopic”. Allergic rhinitis was defined as documentation by a health care provider in the participant’s medical record.

### Laboratory data

Peripheral blood samples were collected at the 13 year visit. The blood was collected in sterile heparinized tubes, kept at room temperature, and then processed the day of collection. Total IgE was measured by fluoroenzyme immunoassays using an automated instrument (Unicap 100; Pharmacia and Upjohn Diagnostics, Kalamazoo, Mich). The sensitivity for detection of total IgE was 2 kU/L [28–29]. Peripheral blood eosinophil numbers were measured by standard methods [29].

### Pulmonary function testing

Spirometry was performed using the Jaeger MasterScope system (Jaeger-Toennies GmbH, Hoechberg, Germany) according to protocols described by the Childhood Asthma Research and Education (CARE) Network [27,30]. The family was instructed to give the child their prescribed asthma medications but to hold albuterol and caffeinated food products for 6 hours prior to the annual visit. If the child was ill or taking albuterol for symptoms, the visit was rescheduled. Fractional exhaled nitric oxide (FeNO) was measured as reported previously[31] using the NIOX system (Aerocrine, Stockholm, Sweden) according to American Thoracic Society online measurement standards adapted for children [32]. The expiratory flow rate was 0.05 L/s. Exhalation times were at least 6 seconds with a 2-second analysis period. Children were required to have 3 measurements within 10% or 2 measurements within 5% for acceptability. Measurements were made before the performance of spirometry [31].

### Statistical methods

Descriptive statistics are reported as means (standard deviations [SD]) for continuous and percentages for categorical variables. Analysis of variance (ANOVA) was used to compare baseline descriptive continuous variables among the groups; Fisher’s Exact tests were used for categorical variables. Before adjustment for confounders, the study was powered to detect a 50 ± 60 μm difference in carotid IMT values with a β = 0.9 and α = 0.05 with a total of 64 participants. A multivariate linear regression model was used to assess the association of carotid IMT and asthma/atopic status, adjusting for biologic confounders. A series of *a priori* models were created by adding potential known biological confounders into each model. Model 1: unadjusted, Model 2: adjusted for age, gender and race, Model 3: fully adjusted, Model 2 + systolic blood pressure and body-mass index. Sensitivity analyses were performed evaluating effect modification by Sex, IgE level and lung function markers. Statistical significance was set at a two-sided p<0.05. We report nominal p values in the context of the study we performed for the group comparisons, all hypotheses tested are reported in the paper Analyses were performed in SAS (Version 9.2, Cary, NC: SAS. Institute Inc.) and R (Version 3.3.0, Vienna, Austria: R Core Team (2016). R: A language and environment for statistical computing. R Foundation for Statistical Computing, Vienna, Austria. URL https://www.R-project.org/).

## Results

### Descriptive characteristics

The 89 participants were mean (SD) 15.3 (0.6) years old, 58% were male, and 89.7% Caucasian. The 28 asthmatics with other atopic disease (As^+^At^+^), 34 asthmatics without other atopic disease (As^+^At^-^) and 15 non-asthmatics with atopic disease (As^-^At^+^) were compared to 12 participants free of asthma and atopic disease (As^-^At^-^). These groups were similar in age, gender, and blood pressure distributions (all p>0.25), but As^+^At^+^ participants tended to have higher body-mass index (p = 0.06), Table 1. By design, our study was enriched with asthmatic participants (p = 0.0006) Table 2.

**Table 1:** Descriptive statistics of included COAST participants.

Characteristic		Neither (A^-^A^-^) (N = 12)	Atopic* only (A-A^+^) (N = 15)	Asthma only (A^+^A^-^) (N = 34)	Atopy* & Asthma (A^+^A^+^) (N = 28)	p value
Age, years (SD)		15.1 (0.3)	15.1 (0.4)	15.3 (0.7)	15.4 (0.6)	0.26
Body-mass index, kg/m^2^ (SD)		20.3 (3.2)	22.2 (4.2)	23.7 (5.9)	24.6 (4.4)	0.06
Male gender, % (N)		50% (6)	60% (9)	53% (18)	68% (19)	0.61
Race, % (N)	Caucasian	92% (11)	100% (15)	91% (31)	86% (24)	0.26
	African-American	0%	0%	6% (2)	14% (4)	0.21
	Asian	8% (1)	0%	3% (1)	0%	0.38
Systolic Blood Pressure, mmHg (SD)		114 (9)	111 (9)	113 (12)	114 (8)	0.70
Diastolic Blood Pressure, mmHg (SD)		64 (5)	63 (4)	65 (5)	64 (4)	0.68
Asthma status at Exam 13	None	100% (12)	100% (15)	26% (9)	14% (4)	N/A
	Intermittent	0%	0%	18% (6)	39% (11)	
	Mild-persistent	0%	0%	47% (16)	29% (8)	
	Moderate-persistent	0%	0%	6% (2)	18% (5)	
	Severe	0%	0%	3% (1)	0%	
Medication Use at year 15	Oral corticosteroid	0%	0%	6% (2)	11% (3)	0.57
	Inhaled corticosteroid	0%	0%	50% (17)	61% (17)	<0.0001
	Leukotriene antagonist	0%	7% (1)	24% (8)	14% (4)	0.21
	Albuterol	17% (2)	0%	74% (25)	86% (24)	<0.0001
	Long acting beta agonist	0%	0%	32% (11)	21% (6)	0.01
Total IgE, median [25^th^, 75^th^]		45 [17,88]	59 [20,132]	83 [53,474]	136 [51,320]	0.08
Blood eosinophil, median [25^th^, 75^th^]		82 [61,128]	198 [112,309]	216 [92,450]	176 [101,280]	0.27
Fractional exhaled nitric oxide, median [25^th^, 75^th^]		13 [12,19]	13 [9,18]	20 [10,38]	18 [11,34]	0.33
Right Carotid Artery Bifurcation μm (SD)		589 (73)	653 (86)	649 (78)	677 (107)	0.04
Right Common Carotid IMT μm (SD)		558 (42)	590 (76)	597 (65)	602 (74)	0.26

Continuous measures are summarized as either mean (standard deviation, SD) or median [25th, 75th], and compared across groups using an analysis of variance. Total IgE, blood eosinophil counts, and exhaled nitric oxide were log-transformed for analysis. Categorical measures are summarized as rates (%) and compared across groups using Fisher’s exact test. Participants may select more than one race category so totals may sum to >100%.

*Physician Diagnosed Atopic Disease (atopic dermatitis and/or allergic rhinitis)

**Table 2:** Descriptive statistics of included COAST participants in this study compared to those not in this study.

Characteristic		Included Participants N = 89	Not Included Participants N = 147	p value
Age, years (SD)		15.3 (0.6)	15 (0)	N/A
Body-mass index, kg/m^2^ (SD)		23.3 (5.0)	22.3 (4.9)	0.17
Male gender, % (N)		58% (52)	54% (79)	0.50
Race % (N)	Caucasian	96% (85)	90% (132)	0.21
	African-American	7% (6)	10% (15)	0.48
	Asian	2% (2)	1% (2)	0.64
Asthma status at Exam 13	None	44% (40)	67% (99)	0.0006
	Intermittent	20% (17)	12% (17)	0.13
	Mild-persistent	28% (24)	14% (20)	0.02
	Moderate-persistent	8% (7)	6% (9)	0.58
	Severe	1% (1)	1% (2)	1
Medication use	Oral corticosteroid	6% (5)	3% (4)	0.30
	Inhaled corticosteroid	38% (34)	18% (26)	0.0006
	Leukotriene antagonist	15% (13)	4% (6)	0.006
	Albuterol	57% (51)	33% (48)	0.0002
	Long acting beta agonist	19% (17)	6% (9)	0.004
Total IgE, median [25^th^, 75^th^]		88 [39, 320]	92 [28, 325]	0.60
Blood eosinophil, median [25^th^, 75^th^]		192 [93, 327]	141 [64, 300]	0.37
Fractional exhaled nitric oxide, median [25^th^, 75^th^]		17 [11, 32]	14 [9, 22]	0.08

Continuous measures are summarized as either mean (SD) or median [25th, 75th], and compared across groups using student’s t-test. Total IgE, blood eosinophil counts, and exhaled nitric oxide were log-transformed for analysis. Categorical measures are summarized as rates (%) and compared across groups using Fisher’s exact test. Participants may select more than one race category so totals may sum to >100%

### Carotid bulb IMT

In unadjusted models, the As^-^At^-^ participants had the lowest RCB IMT values (589 ± 73 μm) compared to both asthmatic groups As^+^At^-^ (649 ± 78 μm, p = 0.05) and A^+^A^+^ (677 ± 107 μm, p = 0.005) (Table 3). In fully adjusted models, the As^-^At^+^ (β = 77.4 μm, 95% confidence intervals [CI], 10.3–144.5 μm, p = 0.02) and both asthmatic groups, As^+^At^-^ (β = 64.2 μm, 95% CI 6.0–122.3 μm, p = 0.03) and As^+^At^+^ (β = 85.8 μm, 95% CI, 24.2–147.4 μm, p = 0.007) had significantly thicker RCB compared to the As^-^At^-^ participants.

**Table 3:** Association of asthma & atopy with subclinical carotid injury.

	Asthma–& Other Atopic Disease* + N = 15		Asthma + & Other Atopic Disease* - N = 34		Asthma + & Other Atopic Disease* + N = 28	
	β value (95% CI)	p value	β value (95% CI)	p value	β value (95% CI)	p value
**Right Carotid Artery Bifurcation Intima-Media Thickness**						
Model 1	64.5 (-5.1–134.1)	0.07	60.2 (0.6–119.8)	**0.05**	88.2 (26.8–149.5)	**0.005**
Model 2	61.7 (-8.0–131.4)	0.08	56.2 (-3.8–116.1)	0.07	79.4 (16.6–142.2)	**0.01**
Model 3	77.4 (10.3–144.5)	**0.02**	64.2 (6.0–122.3)	**0.03**	85.8 (24.2–147.4)	**0.007**
**Right Common Carotid Artery Intima-Media Thickness**						
Model 1	31.9 (-20.1–83.9)	0.23	38.9 (-6.1–84.0)	0.09	44.2 (-2.1–90.5)	0.06
Model 2	30.0 (-22.9–82.8)	0.26	37.9 (-8.0–83.7)	0.10	40.6 (-7.5–88.7)	0.10
Model 3	30.1 (-23.6–83.8)	0.27	34.5 (-12.7–81.7)	0.15	36.2 (-13.7–86.1)	0.15

No Asthma, Atopy (A^-^A^-^) as reference group. Model 1: Unadjusted. Model 2: Adjusted for age, gender, race. Model 3: Fully adjusted, Model 2 + Systolic blood pressure and body-mass index. CI = confidence interval

*Physician-Diagnosed Atopic Disease

### Common carotid artery IMT

In unadjusted models the asthmatic groups (As^+^At^-^ and As^+^At^+^ participants) tended to have higher CCA IMT than As^-^At^-^ participants (β = 38.9 μm, 95% CI -6.1–84.0 μm, p = 0.09 compared to As^+^At^-^ and β = 44.2 μm, 95% CI -2.1–90.5 μm, p = 0.06 As^+^At^+^, respectively. In adjusted models, this trend persisted Table 3.

### Sensitivity analyses

Because total IgE and lung function measures could plausibly confound or mediate an association between asthma and arterial injury[33, 34], we performed sensitivity analyses to assess the effects of both IgE and lung function in our adjusted models. We examined models that included % predicted FEV_1_, FEV_1_/FVC, total IgE and their interactions with asthma/atopic status for both RCCA and RCB. In these models, the size and significance of the group differences were very similar to those in the models without these terms and there was no evidence of interactive effects with the 3 asthmatic/atopic groups compared to the non-asthmatic, non-atopic controls for RCCA or RCB IMT. Based on result from the Swiss Study on Air Pollution and Lung and Heart Disease in Adults (SAPALDIA)[35], we examined effect modification by sex, but did not find a sex by group interaction (p = 0.72).

## Discussion

In a contemporary birth cohort, asthmatic children with or without other atopic disease had thicker RCB and RCCA walls compared to non-asthmatic/non-atopic children, indicating evidence of arterial injury, that is antecedent to the development of CVD. These relationships were identified among adolescents and persisted both after adjustment for several potential biological confounders and in the application of sensitivity analyses. To our knowledge, this is the first report demonstrating the presence of carotid arterial injury in a well-characterized cohort of asthmatic and atopic children. Arterial injury and subsequent atherosclerosis occur more rapidly in the carotid bulb than in the common carotid artery due to the anatomy of the bifurcation and flow disturbances resulting in regions of low and oscillatory shear stress [36–37]. Therefore, the early development of significantly thicker carotid bifurcation IMT in these asthmatic and atopic children identifies an early marker of subclinical arterial changes [36–37]. The differences noted in this study are clinically relevant, since CCA IMT differences of 30–50 μm are associated with approximately 4–6% increases in myocardial infarction and strokes over a decade in adults [38].

Asthma has been associated with increased CVD risk in adults, although the strength of this association has varied widely. Prior reports were from case-control studies and retrospective insurance claims analyses, had differing asthma definitions, or contained homogenous groups of individuals taking older asthma treatments with very few studies investigating subclinical CVD and asthma [13–15,33–34,39–42]. There are limited population level data that describe the association of arterial injury and asthma. Two previous studies have investigated this association in adults and young adults in CVD specific cohorts. In the Atherosclerosis Risk in Communities (ARIC) study, women with adult onset but not childhood onset asthma had increased carotid IMT[34]. Men with either childhood or adult onset asthma did not have increased carotid IMT. In the Atherosclerosis Risk Factors in Male Youngsters and Bruneck studies, allergic conditions such as allergic rhinitis and asthma were associated with thicker carotid or femoral IMT, or more carotid plaque progression in young male and middle-aged adults, respectively[33]. Two reports have examined the association of asthma and carotid IMT in asthmatic children. One demonstrated that children with mild asthma had thicker carotid IMT in a simple, unadjusted analysis; more recently, a report from the SAPALDIA study demonstrated increased carotid IMT in adolescent asthmatic boys but not asthmatic girls [35, 43]. There are less data on atopic conditions and CVD risk. Recently, cross-sectional data from the National Health and Nutrition Examination Survey demonstrated that adults and children with atopic dermatitis have increased risk factors for CVD [44–45]. In the Atherosclerosis Risk Factors in Male Youngsters and Bruneck studies, participants with allergic rhinitis were combined with asthmatics and this group had increased carotid IMT compared to controls [33].

Our study adds significant insight into the association of asthma and CVD. First, we describe the associations of asthma, atopy, and arterial injury in adolescents. Atopic conditions have been associated with CVD risk factors, but prior studies have not reported the effect of atopic conditions with and without asthma on carotid injury. Second, while our sample size is modest, our report is one of the largest studies to date to investigate arterial injury with asthma and atopy. The Atherosclerosis Risk Factors in Male Youngsters study excluded asthmatics and female participants; the Bruneck study had 20 participants with asthma (2.4%); in SAPALDIA, there were 28 asthmatics (19 male, 9 female) [33,35]. Adolescents with and without asthma are ideally suited for studying early arterial injury as their physiology is less confounded by the comorbidities of aging which cause arterial injury and increase CVD risk. Indeed, prior studies included participants with a high prevalence of traditional CVD risk factors. In the COAST study, participants were healthy children free from diabetes mellitus and hypertension and the systolic blood pressures between the non-atopic, non-asthmatic group did not differ from the asthmatic and atopic groups (p = 0.26). Third, this is the first study to investigate the association of asthma, atopy, and carotid IMT in an asthma-specific cohort, with rigorous definitions of the exposure variables. Similar to two previous but limited reports, we found increased arterial injury among early onset asthmatics. Prior studies in adults demonstrated increased arterial injury and CVD events with late-onset asthma; however, few studies have examined the CVD association with early onset asthma and atopic conditions [12,34,41,42]. Our study supports previous reports of arterial injury in early onset asthmatics and suggests that both the early and late onset asthma phenotypes may increase CVD risk.

Early arterial injury occurs in children with other chronic inflammatory diseases such as systemic lupus erythematosus, juvenile idiopathic arthritis and human immunodeficiency virus and predicts higher CVD risks throughout life [46–48]. Asthma is a heterogeneous inflammatory syndrome with different phenotypes each with unique pathophysiology, and the previously reported inconsistent effect sizes and directions for the association of asthma and CVD in prior investigations may be a reflection of this [49]. Atopic dermatitis and allergic rhinitis are also chronic inflammatory disorders that commonly co-exist with childhood asthma [50].

The mechanism(s) by which inflammation in asthma and atopic disease can result in arterial injury and increased CVD risk have not been clarified. Childhood asthma is often associated with a disturbed balance in T-helper cell polarization. T-helper cell polarization occurs primarily in a Type 1 (T_1_) or Type 2 (T_2_) inflammatory profile and childhood asthma and atopy demonstrate a skewed T_2_ pattern of inflammation. T_2_ high asthma is accompanied by elevated levels of IL-4, a pro-atherogenic cytokine that activates the vascular endothelium leading to recruitment and activation of mononuclear cells, increased cellular adhesion molecule expression, and stimulation of 15-lipo-oxygenase production, which can oxidize low-density lipoproteins [51–52]. In addition, mast cells play a central role in asthma and atopic conditions; these mast cells produce the cysteinyl leukotrienes (C_4_, D_4_ and E_4_) and induce acute and chronic inflammation [53]. Animal studies have demonstrated that atherosclerotic plaques contain elevated levels of leukotrienes and that blockade of leukotriene B_4_ reduces monocyte recruitment and atheroma progression [54–55]. Thus, there may be several mechanisms by which the inflammation of early onset asthma and atopy result in arterial injury and increased CVD risk.

## Limitations

Participants from the COAST cohort without asthma or atopic disease served as controls; however, these individuals may be prone to a misclassification bias. To be enrolled in the COAST cohort, children were determined to be at high risk for developing asthma or atopic diseases and therefore some of these children may go on to develop asthma/atopy in the future as they age and/or harbor a subclinical physiology similar to the asthmatic/atopic participants. Nevertheless, this misclassification bias would have influenced our results toward the null. At the time of this study, there were two COAST participants in the control groups that were not diagnosed with asthma at the age 13 year old asthma assessment, but had used albuterol in the past year. These individuals may have been misclassified as not asthmatic, but again, this would bias the results toward the null. This study is observational and the associations we identified do not imply causation. This study was performed in children who were free of many of the CVD confounders that accrue with age. We did not have lipid parameters available for adjustment, however in our full models we adjusted for body mass index which has found to be a sensitive predictor of dyslipidemia in children [56]. Although we adjusted for known biologic confounders, there may have been unmeasured confounders that could have resulted in residual confounding. Although our sensitivity analyses may have been somewhat underpowered, we were able to identify a strong, consistent effect using a modest sample size that was robust to covariate adjustment and that corroborate results found in prior investigations therefore reinforcing the validity of our results.

## Conclusions

Even at a young age, children with asthma and other atopic disease have increased risk of subclinical arterial injury compared to children without asthma or atopy. Given the high prevalence of childhood asthma and atopic conditions, further investigations to confirm this association and elucidate underlying mechanisms are needed.

## Disclosures

Dr. Stein: Inventor, Ultrasonic Apparatus and Method for Providing Quantitative Indication of Risk of Coronary Heart Disease, Patent # US 6,730,0235 B2. Assignee: Wisconsin Alumni Research Foundation, Madison, WI. Filed November 7, 2002. Awarded May 4, 2004. It is used to estimate carotid arterial age based on carotid wall thickness measures. Not used in this study.

## Supporting information

S1 File (XLSX)

## Abbreviations

ARIC: Atherosclerosis Risk in Communities
CI: confidence intervals
COAST: Childhood Origins of Asthma
CVD: cardiovascular disease
IMT: intima-media thickness
NHLBI: National Heart Lung and Blood Institute
RCB: right carotid bifurcation
RCCA: right common carotid
SD: standard deviation
